# 3-Methyl-1-{(*E*)-[1-(4-methyl­pyridin-2-yl)ethyl­idene]amino}­thio­urea: crystal structure and Hirshfeld surface analysis

**DOI:** 10.1107/S2056989018001305

**Published:** 2018-01-31

**Authors:** Lee Chin Lai, Che Nursarah Binti Che Abdul Rahman, M. Ibrahim M. Tahir, Thahira B. S. A. Ravoof, Mukesh M. Jotani, Edward R. T. Tiekink

**Affiliations:** a Department of Chemistry, Faculty of Science, Universiti Putra Malaysia, 43400 UPM Serdang, Selangor Darul Ehsan, Malaysia; b Department of Physics, Bhavan’s Sheth R. A. College of Science, Ahmedabad, Gujarat 380 001, India; c Research Centre for Crystalline Materials, School of Science and Technology, Sunway University, 47500 Bandar Sunway, Selangor Darul Ehsan, Malaysia

**Keywords:** crystal structure, thio­urea derivative, hydrogen bonding, Hirshfeld surface analysis

## Abstract

The title disubstituted thio­urea derivative, C_10_H_14_N_4_S, features an almost planar imine (*E* configuration, C_3_N) core flanked by thio­urea (CN_2_S) and methyl­pyridyl (C_5_N) residues (each plane has a r.m.s. deviation of the respective fitted atoms of 0.0066 Å). The dihedral angles between the core and the thio­urea and pyridyl residues are 20.25 (8) and 7.60 (9)°, respectively, indicating twists in the mol­ecule; the dihedral angle between the outer planes is 13.62 (7)°. There is an *anti*-disposition of the amine-N—H atoms which allows for the formation of an intra­molecular amine-N—H⋯N(imine) hydrogen bond that closes an *S*(5) loop. In the crystal, amine-N—H⋯N(pyrid­yl) hydrogen bonds lead to zigzag (glide symmetry) supra­molecular chains along the *c-*axis direction. These are connected into a supra­molecular layer propagating in the *bc* plane by thio­amide-N—H⋯S(thione) hydrogen bonds *via* eight-membered thio­amide {⋯HNCS}_2_ synthons.

## Abstract 2

The title mol­ecule is twisted, with the dihedral angles between the imine core (C_3_N) and thio­urea and methyl­pyridyl residues being 20.25 (8) and 7.60 (9)°, respectively. In the crystal, corrugated supra­molecular layers in the *bc* plane are mediated by amine-N—H⋯N(pyrid­yl) and thio­amide-N—H⋯S(thione) hydrogen bonds.

## Chemical context   

Thio­semicarbazones (TSCs) are thio­urea derivatives that form versatile ligands containing mixed hard–soft, nitro­gen–sulfur donor atoms. TSC and its derivatives have attracted considerable attention due to their promising biological applications, especially in the realm of anti-tumour (Hussein *et al.*, 2015 ▸), anti-viral (Easmon *et al.*, 1992 ▸), anti-malarial (Kumar *et al.*, 2014 ▸), anti-fungal (Lobana *et al.*, 2017 ▸), anti-bacterial (Khan & Asiri, 2018 ▸) and anti-parasitic (Njogu & Chibale, 2013 ▸) activities. Their biological potential has been found to be enhanced by the addition of alkyl groups at the terminal *N*-position (Liberta and West, 1992 ▸). In fact, a thio­semi­carbazone drug, methisazone (*N*-methyl­isatin β-thio­semi­carbazone) was reported as an anti-viral agent by McNeill in 1972 (McNeill, 1972 ▸) and field trials for methisazone as a prophylactic agent against smallpox were carried out in West Pakistan between 1964 and 1970 (Heiner *et al.*, 1971 ▸). More recently, phase I and phase II clinical trials were conducted for triapine (3-amino­pyridine­carbaldehyde thio­semicarbazone) in untreated patients with advanced-stage cervical cancer where triapine showed an inhibition of ribonucleotide reductase and thus enhanced the radiochemosensitivity by prolonging DNA repair time (Kunos & Sherertz, 2014 ▸). With this inter­est and as a part of on-going investigations on a series of thio­semi­carbazone Schiff bases and their transition metal complexes, the title compound, namely the *N*-methyl thio­semicarbazone derived from 2-acetyl-4-methyl pyridine, (I), was synthesized. Herein, its crystal and mol­ecular structures along with an analysis of its Hirshfeld surface and fingerprint plots are reported.
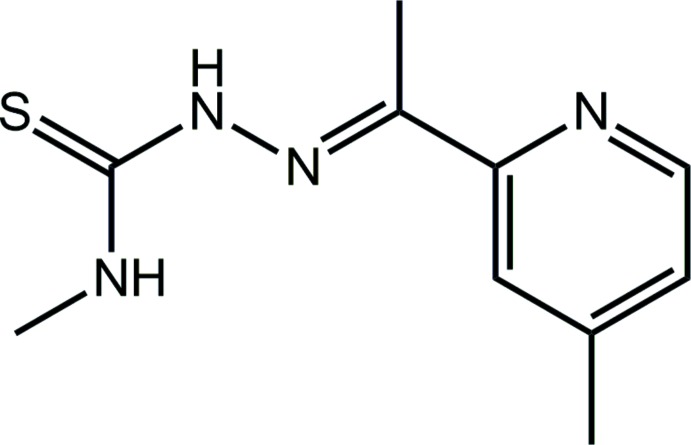


## Structural commentary   

**Figure 1:**
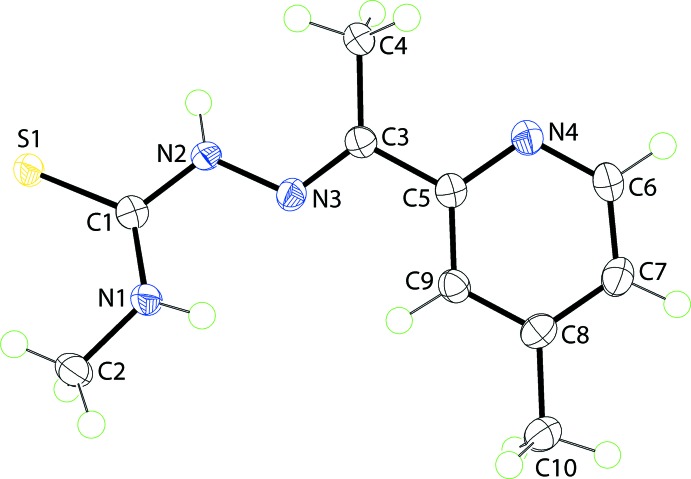
The mol­ecular structure of (I) showing the atom-labelling scheme and displacement ellipsoids at the 70% probability level.

The mol­ecular structure of (I), Fig. 1 ▸, comprises three distinct almost planar residues, namely the thio­urea (C1,N1,N2,S1), central imine (C3,C4,C5,N3) and methyl­pyridyl (N4,C5–C10) residues, coincidentally each with the r.m.s. deviation of the respective fitted atoms being 0.0066 Å. Twists in the mol­ecule are apparent about the N2—N3 and C3—C5 bonds as seen in the values of the C1—N2—N3—C3 and C4—C3—C5—C9 torsion angles of −167.44 (13) and 171.34 (13)°, respectively. This is reflected in the dihedral angles between the mean planes through the central and each of the thio­urea and methyl­pyridyl residues of 20.25 (8) and 7.60 (9)°, respectively; the dihedral angle between the outer planes is 13.62 (7)°. The configuration about the C3=N3 imine bond [1.2872 (19) Å] is *E*. The mol­ecule in (I) features an *anti*-disposition of the amine-N—H atoms, which facilitates the formation of an intra­molecular amine-N1—H⋯N3(imine) hydrogen bond to close an *S*(5) loop, Table 1 ▸. The methyl groups lie to opposite sides of the mol­ecule and can also be described as being *anti* to one another.

## Supra­molecular features   

**Figure 2:**
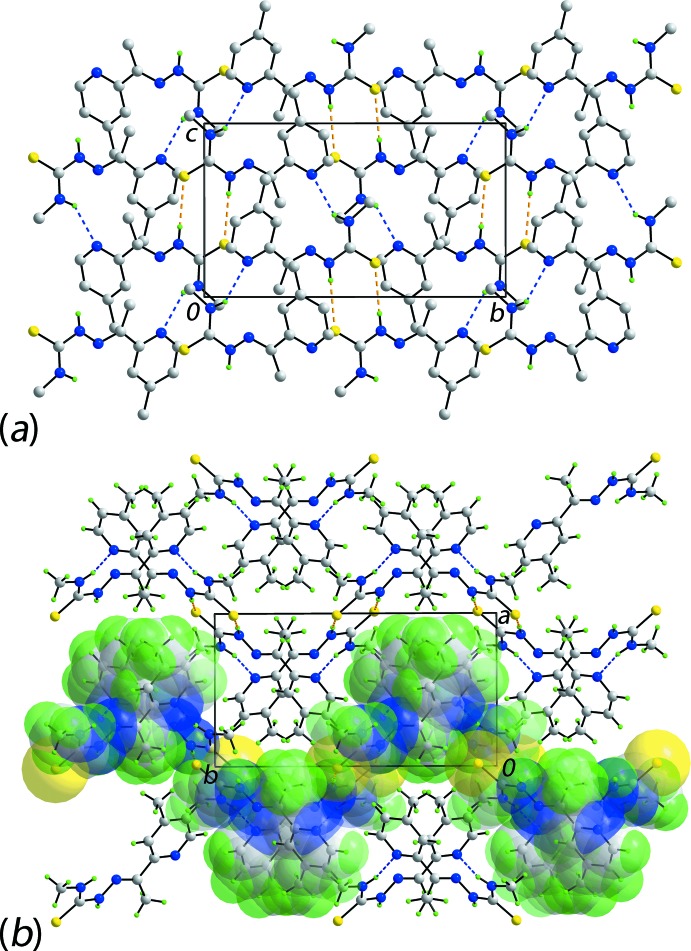
Mol­ecular packing in (I): (*a*) a view of the supra­molecular layer propagating normal to the *a*-axis direction sustained by thio­amide-N—H⋯S(thione) and amine-N—H⋯N(pyrid­yl) hydrogen bonds shown as orange and blue dashed lines, respectively. Non-participating hydrogen atoms have been omitted for reasons of clarity, and (*b*) a view of the unit-cell contents shown in projection down the *c* axis. One layer is highlighted in space-filling mode to emphasize the jagged topology.

The most prominent feature of the mol­ecular packing is the formation of eight-membered thio­amide {⋯HNCS}_2_ synthons owing to the formation of thio­amide-N2—H⋯S1(thione) hydrogen bonds between centrosymmetrically related mol­ecules, Table 1 ▸. These serve to link zigzag (glide symmetry) supra­molecular chains, along the *c-*axis direction and sustained by amine-N1—H⋯N4(pyrid­yl) hydrogen bonds, into a supra­molecular layer propagating in the *bc* plane, Fig. 2 ▸ *a*. Additional stabilization of the layers is afforded by methyl-C—H⋯π(pyrid­yl) inter­actions, Table 1 ▸. Layers stack along the *a* axis without directional inter­actions between them, Fig. 2 ▸ *b*.

## Analysis of the Hirshfeld surfaces   

**Figure 3:**
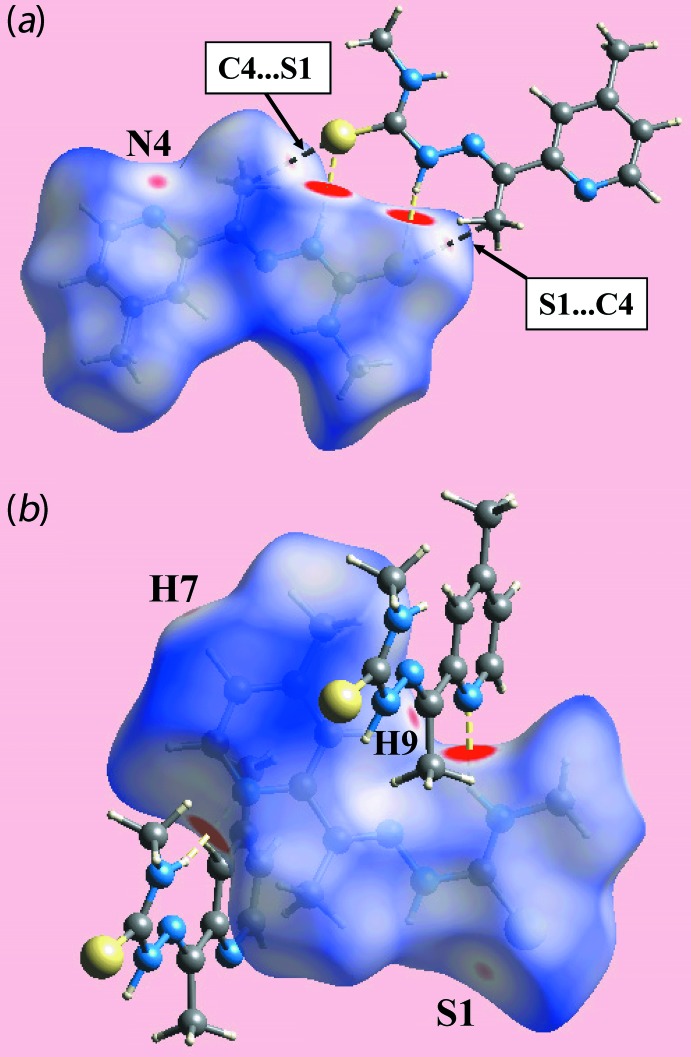
Two views of the Hirshfeld surface mapped over *d*_norm_ for (I) in the range −0.110 to +1.348 au, highlighting N—H⋯N and N—H⋯S hydrogen bonds through yellow dashed lines and short inter­atomic C⋯S/S⋯C contacts through black dashed lines.

**Figure 4:**
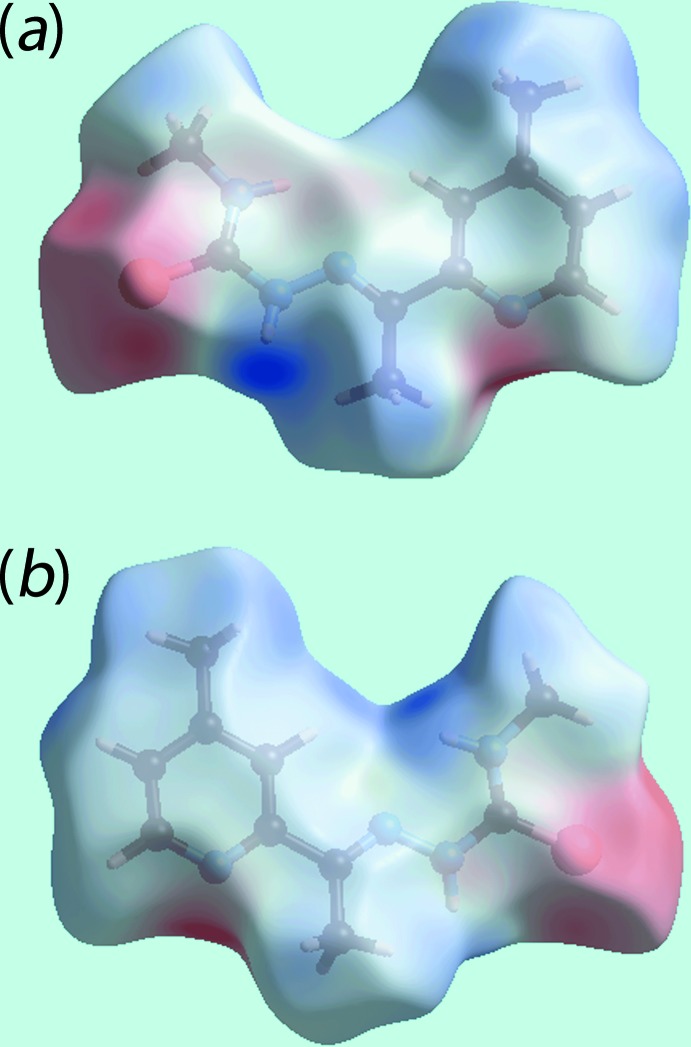
Two views of the Hirshfeld surface mapped over the electrostatic potential for (I) in the range −0.103 to +0.104 au. The red and blue regions represent negative and positive electrostatic potentials, respectively.

The Hirshfeld surface calculations were performed according to recent work on a related organic mol­ecule (Tan *et al.*, 2017 ▸) and serve to provide more detailed information on the influence of inter­molecular inter­actions in the crystal. The dominant N—H⋯S and N—H⋯N hydrogen-bonding interactions in the structure of (I) are viewed as bright-red spots near the respective donor and acceptor atoms on the Hirshfeld surfaces mapped over *d*_norm_ shown in Fig. 3 ▸. The diminutive red spots near the pyridyl-N4 and -H9 atoms indicate the presence of inter­molecular C—H⋯N inter­actions. In addition to the above, the crystal also features comparatively weak inter­molecular C—H⋯S inter­actions and short inter­atomic C⋯S/S⋯C contacts, Table 2 ▸, viewed as faint-red spots in Fig. 3 ▸. The Hirshfeld surfaces mapped over electrostatic potential shown in Fig. 4 ▸ represent the donors and acceptors of inter­molecular inter­actions with blue and red regions corresponding to positive and negative electrostatic potentials, respectively.

**Figure 5:**
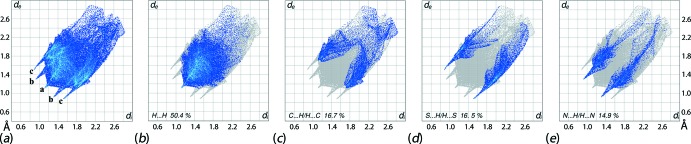
(*a*) The full two-dimensional fingerprint plot for (I) and fingerprint plots delineated into (*b*) H⋯H, (*c*) C⋯H/H⋯C, (*d*) S⋯H/H⋯S and (*e*) N⋯H/H⋯N contacts.

The overall two-dimensional fingerprint plot for (I), showing the key inter­atomic contacts, is illustrated in Fig. 5 ▸ *a*; fingerprint plots delineated (McKinnon *et al.*, 2007 ▸) into H⋯H, C⋯H/H⋯C, S⋯H/H⋯S and N⋯H/H⋯N contacts are shown in Fig. 5 ▸ *b*–*e*. The percentage contributions from the different inter­atomic contacts to the Hirshfeld surface are summarized in Table 3 ▸. A spike at *d*_e_ + *d*_i_ ∼2.2 Å with the label ‘a’ in the middle of the plot and those around it at *d*_e_ + *d*_i_ ∼2.2 and 2.4 Å, labelled with ‘b′ and ‘c′, in the plot of Fig. 5 ▸ *a* indicate the presence of the short inter­atomic H2*A*⋯H4*B* contact (Table 2 ▸) and inter­molecular N—H⋯N and N—H⋯S hydrogen bonds (Table 1 ▸), respectively. The significant contribution of 16.7% from C⋯H/H⋯C contacts to the Hirshfeld surface of (I) is the result of the short C3⋯H4*A* contact (Table 2 ▸) and C—H⋯π inter­action (Table 1 ▸), viewed as a pair of very short peaks at *d*_e_ + *d*_i_ ∼2.8 Å and the parabolic distribution of points around *d*_e_ + *d*_i_ ∼2.9 Å, respectively. The points related to the most prominent inter­layer contact, *i.e*. S1⋯H7 (Table 2 ▸), are merged within the plot delineated into S⋯H/H⋯S contacts (Fig. 5 ▸ *d*) due to the presence of N—H⋯S hydrogen bonds. The contribution of 0.6% from C⋯S/S⋯C contacts to the Hirshfeld surfaces of (I) indicate the presence of the short C4⋯S1 contact listed in Table 2 ▸. The other inter­atomic contacts summarized in Table 3 ▸ having large inter­atomic separations have a negligible effect on the packing.

## Database survey   

Reflecting the inter­est in this class of compounds, there are no fewer than 16 structures related to (I) included in the Cambridge Structural Database (Version 5.38; Groom *et al.*, 2016 ▸), *i.e*. that are neutral and feature N1-bound alkyl or aryl group and a C3-bound pyridyl ring; the C4-bound methyl group is common to all structures. The most closely related structure to (I), *i.e.* with an unsubstituted 2-pyridyl ring at the C3-position, has been described three times, being originally reported in 1999 (Bermejo *et al.*, 1999 ▸). Most structures feature N1-bound aryl rings, and all feature an *anti*-disposition of the N—H groups.

## Synthesis and crystallization   

All chemicals were of analytical grade and were used without any further purification. 2-Acetyl-4-methyl pyridine (0.68 g, 0.005 mol) in absolute ethanol (40 ml) was dissolved and added to 4-methyl-3-thio­semicarbazide (0.52 g, 0.005 mol) dissolved in absolute ethanol (40 ml). The mixture was then heated in a water bath for 10 mins with constant and vigorous stirring until the volume reduced to 30 ml. The product that formed was filtered off, washed with cold ethanol and dried in a desiccator over anhydrous silica gel. Brown platy crystals suitable for single crystal X-ray diffraction analysis were obtained by recrystallization with absolute ethanol as solvent. M.pt: 468.8–470.1 K. IR (cm^−1^): 3274 ν(N—H), 1589 ν(C=N), 1118 ν(N—N), 1045 ν(C=S). MS (*m*/*z*): 222.

## Refinement   

Crystal data, data collection and structure refinement details are summarized in Table 4 ▸. The carbon-bound H atoms were placed in calculated positions (C—H = 0.95–0.98 Å) and were included in the refinement in the riding model approximation, with *U*_iso_(H) set to 1.2–1.5*U*_eq_(C). The nitro­gen-bound H atoms were located in a difference-Fourier map but were refined with a distance restraint of N—H = 0.88±0.01 Å, and with *U*_iso_(H) set to 1.2*U*_eq_(N).

## Supplementary Material

Crystal structure: contains datablock(s) I, global. DOI: 10.1107/S2056989018001305/hb7729sup1.cif

Structure factors: contains datablock(s) I. DOI: 10.1107/S2056989018001305/hb7729Isup2.hkl

CCDC reference: 1818317

Additional supporting information:  crystallographic information; 3D view; checkCIF report

## Figures and Tables

**Table 1:** Hydrogen-bond geometry (Å, °) *Cg*1 is the centroid of the (N4,C5–C9) ring.

*D*—H⋯*A*	*D*—H	H⋯*A*	*D*⋯*A*	*D*—H⋯*A*
N1—H1*N*⋯N3	0.88 (2)	2.23 (2)	2.6148 (18)	106 (1)
N1—H1*N*⋯N4^i^	0.88 (2)	2.33 (2)	3.0714 (18)	142 (1)
N2—H2*N*⋯S1^ii^	0.87 (1)	2.55 (1)	3.3955 (13)	168 (2)
C10—H10*A*⋯*Cg*1^i^	0.98	2.89	3.7546 (18)	147

Symmetry codes: (i) 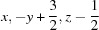; (ii) 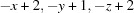.

**Table 2:** Summary of short inter­atomic contacts (Å) in (I)

Contact	Distance	Symmetry operation
H2*A*⋯H4*B*	2.23	2 − *x*, − 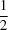 + *y*, 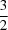 − *z*
H9⋯N4	2.54	*x*, 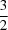 − *y*, − 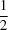 + *z*
H7⋯S1	2.83	−1 + *x*, 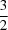 − *y*, − 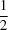 + *z*
C3⋯H4*A*	2.84	*x*, 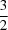 − *y*, − 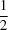 + *z*
C4⋯S1	3.4545 (15)	2 − *x*, 1 − *y*, 2 − *z*

**Table 3:** Relative percentage contributions of close contacts to the Hirshfeld surface of (I)

H⋯H	50.4
C⋯H/H⋯C	16.7
S⋯H/H⋯S	16.5
N⋯H/H⋯N	14.9
C⋯C	0.7
C⋯S/S⋯C	0.6
S⋯N/N⋯S	0.2

**Table 4:** Experimental details

Crystal data	
Chemical formula	C_10_H_14_N_4_S
*M*_r_	222.31
Crystal system, space group	Monoclinic, *P*2_1_/*c*
Temperature (K)	100
*a*, *b*, *c* (Å)	8.8108 (3), 14.9044 (4), 9.3583 (3)
β (°)	113.391 (4)
*V* (Å^3^)	1127.93 (7)
*Z*	4
Radiation type	Cu *K*α
μ (mm^−1^)	2.33
Crystal size (mm)	0.15 × 0.13 × 0.03

Data collection	
Diffractometer	Rigaku Oxford Diffraction Gemini E
Absorption correction	Multi-scan (*CrysAlis PRO*; Agilent, 2011 ▸)
*T*_min_, *T*_max_	0.852, 1.000
No. of measured, independent and observed [*I* > 2σ(*I*)] reflections	21767, 2184, 1995
*R*_int_	0.034
(sin θ/λ)_max_ (Å^−1^)	0.614

Refinement	
*R*[*F* ^2^ > 2σ(*F* ^2^)], *wR*(*F* ^2^), *S*	0.035, 0.097, 1.02
No. of reflections	2184
No. of parameters	145
No. of restraints	2
H-atom treatment	H atoms treated by a mixture of independent and constrained refinement
Δρ_max_, Δρ_min_ (e Å^−3^)	0.41, −0.23

Computer programs: *CrysAlis PRO* (Agilent, 2011 ▸), *SHELXS97* (Sheldrick, 2008 ▸), *SHELXL2014* (Sheldrick, 2015 ▸), *ORTEP-3 for Windows* (Farrugia, 2012 ▸), *DIAMOND* (Brandenburg, 2006 ▸) and *publCIF* (Westrip, 2010 ▸).

## supplementary crystallographic information

### Crystal data

**Table:**

C_10_H_14_N_4_S	*F*(000) = 472
*M**_r_* = 222.31	*D*_x_ = 1.309 Mg m^−^^3^
Monoclinic, *P*2_1_/*c*	Cu *K*α radiation, λ = 1.5418 Å
*a* = 8.8108 (3) Å	Cell parameters from 9894 reflections
*b* = 14.9044 (4) Å	θ = 3.0–71.2°
*c* = 9.3583 (3) Å	µ = 2.33 mm^−^^1^
β = 113.391 (4)°	*T* = 100 K
*V* = 1127.93 (7) Å^3^	Plate, brown
*Z* = 4	0.15 × 0.13 × 0.03 mm

### Data collection

**Table:**

Rigaku Oxford Diffraction Gemini E diffractometer	2184 independent reflections
Radiation source: Enhance X-ray source	1995 reflections with *I* > 2σ(*I*)
Mirror monochromator	*R*_int_ = 0.034
Detector resolution: 16.1952 pixels mm^-1^	θ_max_ = 71.3°, θ_min_ = 5.5°
ω scan	*h* = −10→10
Absorption correction: multi-scan (CrysAlis PRO; Agilent, 2011)	*k* = −18→17
*T*_min_ = 0.852, *T*_max_ = 1.000	*l* = −11→11
21767 measured reflections	

### Refinement

**Table:**

Refinement on *F*^2^	Primary atom site location: structure-invariant direct methods
Least-squares matrix: full	Hydrogen site location: mixed
*R*[*F*^2^ > 2σ(*F*^2^)] = 0.035	H atoms treated by a mixture of independent and constrained refinement
*wR*(*F*^2^) = 0.097	*w* = 1/[σ^2^(*F*_o_^2^) + (0.063*P*)^2^ + 0.4965*P*] where *P* = (*F*_o_^2^ + 2*F*_c_^2^)/3
*S* = 1.02	(Δ/σ)_max_ = 0.001
2184 reflections	Δρ_max_ = 0.41 e Å^−^^3^
145 parameters	Δρ_min_ = −0.23 e Å^−^^3^
2 restraints	

### Special details

**Table:**

Geometry. All esds (except the esd in the dihedral angle between two l.s. planes) are estimated using the full covariance matrix. The cell esds are taken into account individually in the estimation of esds in distances, angles and torsion angles; correlations between esds in cell parameters are only used when they are defined by crystal symmetry. An approximate (isotropic) treatment of cell esds is used for estimating esds involving l.s. planes.

### Fractional atomic coordinates and isotropic or equivalent isotropic displacement parameters (Å^2^)

**Table:**

	*x*	*y*	*z*	*U*_iso_*/*U*_eq_	
S1	1.01197 (4)	0.43326 (2)	0.80392 (4)	0.01660 (14)	
N1	0.78625 (16)	0.52040 (9)	0.56429 (14)	0.0167 (3)	
H1N	0.728 (2)	0.5689 (9)	0.527 (2)	0.020*	
N2	0.85960 (15)	0.58621 (8)	0.80403 (14)	0.0151 (3)	
H2N	0.908 (2)	0.5811 (12)	0.9041 (11)	0.018*	
N3	0.73527 (15)	0.64746 (8)	0.73280 (14)	0.0151 (3)	
N4	0.57917 (15)	0.85680 (9)	0.79338 (14)	0.0169 (3)	
C1	0.87633 (17)	0.51659 (10)	0.71665 (16)	0.0142 (3)	
C2	0.7888 (2)	0.45156 (11)	0.45507 (18)	0.0206 (3)	
H2A	0.9018	0.4289	0.4860	0.031*	
H2B	0.7508	0.4772	0.3502	0.031*	
H2C	0.7155	0.4022	0.4554	0.031*	
C3	0.73564 (17)	0.72169 (10)	0.80323 (16)	0.0137 (3)	
C4	0.86453 (18)	0.75205 (10)	0.95558 (17)	0.0176 (3)	
H4A	0.8263	0.7403	1.0389	0.026*	
H4B	0.8841	0.8165	0.9508	0.026*	
H4C	0.9676	0.7192	0.9768	0.026*	
C5	0.59425 (17)	0.78267 (10)	0.71764 (16)	0.0144 (3)	
C6	0.45183 (19)	0.91141 (11)	0.71570 (19)	0.0201 (3)	
H6	0.4387	0.9638	0.7675	0.024*	
C7	0.33818 (18)	0.89648 (11)	0.56488 (18)	0.0198 (3)	
H7	0.2515	0.9381	0.5154	0.024*	
C8	0.35303 (17)	0.81950 (10)	0.48712 (16)	0.0168 (3)	
C9	0.48424 (17)	0.76211 (10)	0.56638 (16)	0.0153 (3)	
H9	0.4989	0.7089	0.5175	0.018*	
C10	0.23240 (19)	0.79716 (11)	0.32435 (18)	0.0205 (3)	
H10A	0.2929	0.7874	0.2576	0.031*	
H10B	0.1546	0.8470	0.2830	0.031*	
H10C	0.1714	0.7426	0.3267	0.031*	

### Atomic displacement parameters (Å^2^)

**Table:**

	*U*^11^	*U*^22^	*U*^33^	*U*^12^	*U*^13^	*U*^23^
S1	0.0203 (2)	0.0138 (2)	0.0143 (2)	0.00414 (13)	0.00545 (15)	0.00082 (13)
N1	0.0217 (6)	0.0131 (6)	0.0134 (6)	0.0032 (5)	0.0050 (5)	0.0000 (5)
N2	0.0174 (6)	0.0140 (6)	0.0111 (6)	0.0029 (5)	0.0028 (5)	0.0006 (5)
N3	0.0158 (6)	0.0148 (6)	0.0144 (6)	0.0029 (5)	0.0057 (5)	0.0017 (5)
N4	0.0178 (6)	0.0160 (7)	0.0171 (6)	0.0018 (5)	0.0072 (5)	−0.0005 (5)
C1	0.0152 (7)	0.0125 (7)	0.0161 (7)	−0.0017 (5)	0.0073 (5)	0.0008 (5)
C2	0.0278 (8)	0.0167 (8)	0.0158 (7)	0.0015 (6)	0.0072 (6)	−0.0027 (6)
C3	0.0158 (7)	0.0143 (7)	0.0126 (7)	0.0000 (5)	0.0073 (5)	0.0008 (5)
C4	0.0193 (7)	0.0141 (7)	0.0166 (7)	0.0020 (6)	0.0042 (6)	−0.0023 (6)
C5	0.0161 (7)	0.0134 (7)	0.0156 (7)	−0.0002 (5)	0.0082 (6)	0.0013 (5)
C6	0.0216 (7)	0.0157 (8)	0.0228 (8)	0.0035 (6)	0.0086 (6)	−0.0022 (6)
C7	0.0174 (7)	0.0185 (8)	0.0223 (8)	0.0043 (6)	0.0067 (6)	0.0022 (6)
C8	0.0162 (7)	0.0189 (8)	0.0158 (7)	0.0000 (6)	0.0069 (6)	0.0033 (6)
C9	0.0182 (7)	0.0143 (7)	0.0149 (7)	0.0005 (6)	0.0081 (6)	0.0000 (5)
C10	0.0204 (7)	0.0221 (8)	0.0166 (7)	0.0028 (6)	0.0050 (6)	0.0028 (6)

### Geometric parameters (Å, º)

**Table:**

S1—C1	1.6920 (15)	C4—H4A	0.9800
N1—C1	1.3295 (18)	C4—H4B	0.9800
N1—C2	1.4547 (19)	C4—H4C	0.9800
N1—H1N	0.873 (9)	C5—C9	1.396 (2)
N2—C1	1.3649 (19)	C6—C7	1.386 (2)
N2—N3	1.3776 (17)	C6—H6	0.9500
N2—H2N	0.864 (9)	C7—C8	1.392 (2)
N3—C3	1.2872 (19)	C7—H7	0.9500
N4—C5	1.3474 (19)	C8—C9	1.392 (2)
N4—C6	1.342 (2)	C8—C10	1.508 (2)
C2—H2A	0.9800	C9—H9	0.9500
C2—H2B	0.9800	C10—H10A	0.9800
C2—H2C	0.9800	C10—H10B	0.9800
C3—C5	1.4923 (19)	C10—H10C	0.9800
C3—C4	1.4971 (19)		
			
C1—N1—C2	123.66 (13)	H4A—C4—H4C	109.5
C1—N1—H1N	117.9 (13)	H4B—C4—H4C	109.5
C2—N1—H1N	118.3 (13)	N4—C5—C9	122.66 (13)
C1—N2—N3	117.92 (12)	N4—C5—C3	116.91 (12)
C1—N2—H2N	117.3 (12)	C9—C5—C3	120.42 (13)
N3—N2—H2N	122.4 (12)	N4—C6—C7	124.37 (14)
C3—N3—N2	118.81 (12)	N4—C6—H6	117.8
C5—N4—C6	116.62 (12)	C7—C6—H6	117.8
N1—C1—N2	116.71 (13)	C6—C7—C8	118.98 (14)
N1—C1—S1	123.75 (11)	C6—C7—H7	120.5
N2—C1—S1	119.50 (11)	C8—C7—H7	120.5
N1—C2—H2A	109.5	C7—C8—C9	117.28 (13)
N1—C2—H2B	109.5	C7—C8—C10	122.34 (14)
H2A—C2—H2B	109.5	C9—C8—C10	120.38 (14)
N1—C2—H2C	109.5	C8—C9—C5	120.08 (14)
H2A—C2—H2C	109.5	C8—C9—H9	120.0
H2B—C2—H2C	109.5	C5—C9—H9	120.0
N3—C3—C5	114.63 (12)	C8—C10—H10A	109.5
N3—C3—C4	126.45 (13)	C8—C10—H10B	109.5
C5—C3—C4	118.89 (12)	H10A—C10—H10B	109.5
C3—C4—H4A	109.5	C8—C10—H10C	109.5
C3—C4—H4B	109.5	H10A—C10—H10C	109.5
H4A—C4—H4B	109.5	H10B—C10—H10C	109.5
C3—C4—H4C	109.5		
			
C1—N2—N3—C3	−167.44 (13)	N3—C3—C5—C9	−6.6 (2)
C2—N1—C1—N2	−179.61 (13)	C4—C3—C5—C9	171.34 (13)
C2—N1—C1—S1	2.5 (2)	C5—N4—C6—C7	−0.5 (2)
N3—N2—C1—N1	9.20 (19)	N4—C6—C7—C8	1.0 (2)
N3—N2—C1—S1	−172.80 (10)	C6—C7—C8—C9	−0.8 (2)
N2—N3—C3—C5	−178.98 (11)	C6—C7—C8—C10	178.57 (15)
N2—N3—C3—C4	3.2 (2)	C7—C8—C9—C5	0.1 (2)
C6—N4—C5—C9	−0.3 (2)	C10—C8—C9—C5	−179.24 (13)
C6—N4—C5—C3	179.94 (13)	N4—C5—C9—C8	0.4 (2)
N3—C3—C5—N4	173.16 (12)	C3—C5—C9—C8	−179.77 (13)
C4—C3—C5—N4	−8.85 (19)		

### Hydrogen-bond geometry (Å, º)

Cg1 is the centroid of the (N4,C5–C9) ring.

**Table:**

*D*—H···*A*	*D*—H	H···*A*	*D*···*A*	*D*—H···*A*
N1—H1*N*···N3	0.88 (2)	2.23 (2)	2.6148 (18)	106 (1)
N1—H1*N*···N4^i^	0.88 (2)	2.33 (2)	3.0714 (18)	142 (1)
N2—H2*N*···S1^ii^	0.87 (1)	2.55 (1)	3.3955 (13)	168 (2)
C10—H10*A*···*Cg*1^i^	0.98	2.89	3.7546 (18)	147

Symmetry codes: (i) *x*, −*y*+3/2, *z*−1/2; (ii) −*x*+2, −*y*+1, −*z*+2.
